# Supplementary material for: Are carbon emissions trading and green financial instruments synergistic? -Comprehensive quantitative research based on content analysis
Source: PLoS One. 2024 Mar 7;19(3):e0298601. doi: 10.1371/journal.pone.0298601 (PMC10919601; doi:10.1371/journal.pone.0298601)
Supplement: S1 Data — (ZIP) [file pone.0298601.s001.zip › Data/地级市Z_F工作报告.zip!/╔╜╢1⁄2/╠⌐░▓╩╨/└┤╫╘╣1⁄2╓┌║┼í╛┬φ┐╦╩2╛▌═°í┐_2016.docx]

各位代表：

　　现在，我代表市人民政府向大会报告工作，请予审议，并请市政协委员和列席会议的同志提出意见。

　　“十二五”时期工作回顾

　　“十二五”时期是我市发展极不平凡的五年。面对复杂严峻的形势，在市委的坚强领导下，全市上下紧紧围绕推进富民强市、建设幸福泰安奋斗目标，稳增长、调结构、促改革、惠民生、防风险，砥砺奋进，扎实工作，改革开放和现代化建设取得显著成绩。

　　——这五年，是我市克服艰难险阻，经济综合实力稳步提升的五年。有效应对国际金融危机、全球贸易增长乏力等不利影响，克服国内“三期叠加”、经济减速换挡、部分行业产能过剩和效益下滑等困难，经济发展稳中有进、稳中向好。预计2015年实现生产总值3240亿元，是2010年的1.6倍，年均增长10%；一般公共预算收入突破200亿元，达到205.3亿元，是2010年的1.76倍，年均增长11.9%，税收占比（同口径）达到73.3%；实现社会消费品零售总额1329亿元，年均增长14.1%；金融机构各项存贷款余额分别达到2704.96亿元和1800.56亿元，年均增长18.7%和19.2%。

　　——这五年，是我市转方式调结构步伐加快，新产业新业态蓬勃发展的五年。预计三次产业结构调整为8.8︰46.2︰45.0。扎实开展千亿斤粮食产能建设和高产创建活动，粮食连年增产丰收。大力实施十四大特色优势产业振兴规划，有机蔬菜、苗木花卉、泰山茶分别发展到35.3万亩、40万亩、3.5万亩，预计奶牛存栏达到27万头。规模以上农业龙头企业、农民合作社、家庭农场分别达到870家、6893家、2436家。东方园林、滕头园林、澳亚牧场、益客食品等一批特色农业园区加快发展。杞农云商、农村淘宝等一批农业电商平台和电商村不断涌现。农业机械化水平达到86%，比2010年提高8个百分点。泰安农高区升级为国家农业科技园区，岱岳区被列为国家级现代农业示范区。

　　大力实施工业转型升级、百亿企业培植、互联网工业等计划，预计规模以上工业企业达到1970家，实现主营业务收入6850亿元，是2010年的1.91倍；主营业务收入过10亿元企业达到116家，比2010年增加63家。泰开集团、瑞星集团、石横特钢、泰丰集团主营业务收入突破百亿。预计新材料、新一代信息技术、新医药产业主营业务收入占比达到13%。新能源装机容量达到480.1兆瓦，我市被列为国家新能源示范城市、资源综合利用“双百工程”示范基地，泰安高新区被列为国家首批分布式光伏发电示范区，新泰市被确定为中德新能源示范市。累计实施技术改造项目4000余个。新增中国驰名商标27个。康平纳、中联水泥、泰山玻纤列入国家智能制造试点示范项目。全面完成了淘汰落后产能和节能减排目标任务。

　　大力实施“103030”工程，预计2015年服务业投资占固定资产投资比重达到54%，税收占比达到48.6%。太阳部落、泰山温泉城、刘老根大舞台、宝泰隆旅游度假区、泰山千古情、大汶口国家考古遗址公园等一批精品旅游文化项目相继建成，“三日游”成效明显，游客满意度大幅提升。预计2015年旅游消费总额达到571.7亿元，年均增长17.2%；旅游业占生产总值比重达到17.65%，比2010年提高5.06个百分点。泰山被授予国家级海峡两岸交流基地、全国旅游服务质量标杆单位，岱岳区、东平县被评为国家级休闲农业与乡村旅游示范县。新发展现代物流园区24个、规模以上物流企业221家，新矿物流中心进入全国物流企业百强。预计2015年电子商务交易额达到600亿元，泰安高新区物流电商园、泰山梦想谷电商产业园、岱岳区青春创业电子商务产业园、新泰跨境电子商务产业园、新易泰物流产业园、肥城鲁中国际电子商务产业园、宁阳泰山电子商务产业园、东平电子商务产业园8个电商园区加快建设。泰山国际会展中心建成使用。信息服务、健康养生、家政服务等新兴服务业发展迅速。

　　——这五年，是我市项目建设力度加大，发展后劲显著增强的五年。坚持把项目建设和招商引资作为主要抓手，实行指挥部推进和重点项目市级领导包保制度，加大高效优质投入。预计固定资产投资累计完成10156.4亿元，是“十一五”的2.41倍，年均增长17.2%。累计实施市级重点建设项目473个，完成投资2152亿元。大力推进产业招商，累计引进总投资过10亿元大项目127个。泰安如意科技时尚产业园、中材玻纤产业园、中国·联合绿色建材产业集聚区、航天特车工业园、肥城现代盐化工园区、润恒现代农副产品冷链物流产业园等加快建设，泰山云谷互联网产业园、神农智谷产业园、泰邦生物科技园、紫晶立方3D打印、智能机器人等新兴产业项目加快推进。中国五矿集团、中国诚通集团、中国重汽集团、中粮集团、澳大利亚博罗石膏、芬兰卡哥特科、新加坡益海嘉里和泺亨集团等一批国内外知名企业落户泰安。

　　——这五年，是我市科技创新能力不断增强，发展动力日益强劲的五年。深入实施创新驱动发展战略，预计全市研究与试验发展经费投入占生产总值比重达到2.28%，比2010年提高0.79个百分点。国家级高新技术企业达到114家，比2010年增加68家；预计高新技术产业产值占工业总产值比重达到26.9%，提高5.63个百分点。85%以上的规模企业与高校院所建立了合作关系，90%以上的大中型骨干企业建立了省级以上技术研发机构。企业重点实验室、工程技术研究中心、科技孵化器等省级以上创新平台达到128家，其中国家级企业技术中心达到4家。康平纳集团获国家科技进步一等奖，岱银集团获国家专利发明金奖。泰安高新区升级为国家级高新技术园区。我市跻身中国城市综合创新能力50强。

　　——这五年，是我市各项改革深入推进，开放型经济突破发展的五年。市县机构改革全面完成，行政审批制度改革不断深化，编制公布了行政审批事项目录清单、行政权力清单和政府部门责任清单。实施商事制度改革，落实“先照后证”“三证合一、一照一码”制度，新登记各类市场主体12.4万户。政府预算体系和透明预算制度基本建立，“营改增”等税制改革和支出管理制度改革扎实推进，公务用车改革启动实施。深化投融资体制改革，政府与社会资本合作取得积极成效。探索建立了“三位一体”国资国企改革架构，组建了国泰民安投资集团，全市国有企业资产总额达到1830亿元，是2010年的2.8倍；市属国有企业累计上缴国有资产收益9.2亿元，是“十一五”的3.8倍。新设立地方金融组织56家，13家异地银行、17家保险机构落户泰安；宁阳、泰山农信社改制为农商行，岱岳、新泰、肥城、东平4家农信社银行化改革实现达标；泰盈科技在美国纳斯达克上市，12家企业在“新三板”挂牌。农村土地承包经营权确权登记颁证工作基本完成，202个村完成农村集体经济产权制度改革，东平县被确定为第二批全国农村改革试验区；13处国有林场改革试点通过国家验收；集体林权配套改革深入推进，新泰市被列为全国集体林业综合改革试验示范区和全国林下经济示范基地县。

　　开放型经济突破发展，累计新批外资项目274个，实际利用外资14.9亿美元，是“十一五”的13.7倍；累计实现进出口总值117.46亿美元，年均增长7.6%，其中出口72.76亿美元,年均增长13.8%；新增进出口获权企业625家，外贸企业达到1465家，我市跻身中国外贸竞争力百强城市。累计完成境外投资5.3亿美元，年均增长38%；境外投资企业达到56家，比2010年增加31家。泰安高新区综合监管区和青岛保税港区（泰安）功能区启动建设。

　　——这五年，是我市城乡面貌焕然一新，生态建设成效显著的五年。预计全市常住人口城镇化率达到56%，比2010年提高5.65个百分点。突出城市发展宜居性和可持续性，编制完成城市空间发展战略研究。泰城高标准实施了片区开发、组团建设和功能提升，“两轴”“两带”纵横贯通，万达城市广场建成运营，天房美郡、中南·财源门、五矿万境水岸等城市综合体加快推进，高铁新区、新华片区、碧霞国际社区等城市片区建设全面铺开；实施了徂徕山汶河景区开发、引水入城及104国道改建、万官大街、泮河大街、泰山火车站广场改造、城市河道治理、道路美化提升等重点工程，新拓展城市发展空间147平方公里，建成区绿化覆盖率达到44%；三合水厂提质改造、供热“汽改水”、第一和第二污水处理厂改造升级、第四污水处理厂建设顺利完成，国电热电联产项目一期建成投产，智能配电网实现全覆盖；新增环保型公交车1366台，实行了“一小时免费·优惠换乘”。加大环境综合整治力度，泰城获得中国人居环境奖城市称号，顺利通过国家卫生城市复审。各县市区坚持产城融合发展，加快县域、镇域经济提升，实施了一大批城镇基础设施项目，发展活力和承载力明显增强。新泰市被确定为全省县域经济科学发展试点县，肥城市被确定为国家主体功能区建设试点示范县。

　　大力实施“蓝天工程”，大气质量在全省内陆城市排名首位。落实最严格水资源管理制度，加大水环境治理力度，我市被列为全国水生态文明城市建设试点，汶河、康王河、东平湖3处湿地被批建为国家湿地公园，徂徕山汶河水利风景区、肥城龙山河水利风景区入选国家水利风景区。实施了大汶河综合开发，形成了长50公里、静态蓄水8000万立方米的生态长廊。治理水土流失面积400平方公里。完成造林110.3万亩，森林覆盖率达到39.5%，成功创建为国家森林城市，泰山、徂徕山入选全国首批“中国森林氧吧”。生态文明乡村行动深入推进，实施农村新型社区集中建设项目431个，建设农房30万户，改造危房8.5万户；建成省级生态区1个、省级以上生态乡镇65个、省级生态村30个；城乡环卫一体化实现全覆盖。

　　——这五年，是我市民生投入最大，群众得到更多实惠的五年。全市累计各项民生支出1011.8亿元，是“十一五”的2.5倍，占公共财政支出的76.5%。预计城镇和农村居民人均可支配收入分别达到28100元和13400元。新增城镇就业34.56万人、农村劳动力转移就业44.96万人，被国务院表彰为全国创业先进城市。累计收缴各项社会保险费555.6亿元，发放各项社会保险待遇454.8亿元。社会养老保险制度实现全覆盖，居民养老保险基础养老金标准由2010年每人每月55元提高到85元；城乡居民医疗保险制度实现并轨，在全省率先建立城镇居民大病医疗保险制度；失业保险标准统一提高到月人均900元；城乡低保标准分别由2010年的月均267.5元、年均1225元，提高到499元和3350元。五保集中和分散供养标准分别由2010年的3325元、2325元，提高到5763元和4550元。建成城市社区老年人日间照料中心176处、农村幸福院887处，养老床位由2010年的1.6万张增加到3.2万张。累计发放公积金贷款52亿元。建设各类保障性安居房10.28万套。完成国家小农水重点县建设、水库除险加固、河道治理、山洪灾害防治、农村饮水安全等各类水利工程2.1万项。累计新改建农村公路3425.1公里，80.48万农村人口饮水安全问题得到解决，村村通柏油（水泥）路、通客车和农村自来水普及率分别达到100%、99.5%和98%。全市23.1万贫困人口顺利脱贫，东平县特困移民工程列为全国避险解困试点。各项强农惠农富农政策得到落实。

　　——这五年，是我市社会事业全面进步，社会治理不断创新的五年。幸福教育大力实施，学前教育三年行动计划有效落实，新改扩建公办幼儿园564处；义务教育薄弱学校办学条件全面改善，5个县市区通过了国家义务教育均衡发展验收；高中段教育普及率和办学水平持续提高；泰安一中新城校区和市特教中心加快建设；现代职业教育体系逐步完善，在全省率先实现国家中职示范校和省级规范化中职学校县市区全覆盖。基层医疗卫生机构综合改革全面完成，14家县级公立医院实施了综合改革试点，基本药物制度实现全覆盖，基本公共卫生服务人均项目经费由2010年的15元提高到40元；重点传染病防控、主要疾病控制和卫生应急工作得到加强；泰安市妇幼保健院新院建成启用；我市荣获全国基层中医药工作先进单位、全国人口和计划生育综合改革示范市称号。实施文化惠民工程，乡镇综合文化站实现全覆盖，建成泰安文化艺术中心；我市被确定为全国文明城市提名城市，泰山区、新泰市、肥城市入选全国文化先进县，东平戴村坝列入世界文化遗产。我市第五次荣获全国双拥模范城称号。体育事业取得新成绩。对口援建新疆岳普湖县和重庆巫溪县工作成效明显。国防动员、人民防空、民兵预备役工作顺利开展。妇女儿童和残疾人保障、老龄工作有新提高。外事侨务、民族宗教、气象、防震、史志、档案等社会事业全面进步。

　　“六五”普法依法治理工作扎实推进。城市社区“一站式”综合服务平台实现全覆盖。我市被确定为全国首批社会组织建设创新示范区。“天网工程”、大数据警务云体系、民生警务平台有效运行，立体化社会治安防控体系日益完善。食品药品市县乡村四级监管体系初步形成。政府应急管理工作得到加强，作用发挥较好。“安如泰山”科学预防体系加快建设，安全生产形势持续稳定。

　　——这五年，是政府作风切实转变，服务效能较大提升的五年。扎实开展党的群众路线教育实践活动和“三严三实”专题教育，认真落实中央八项规定精神，集中开展“庸懒散”专项整治，“四风”和“不严不实”问题得到有效解决。建立政府法律顾问制度，完善政府决策机制，法治政府建设取得新成效。集中整治行政审批“五不”现象，规范中介机构，项目审批效率有较大提高。建立了市级公共资源交易平台。认真开展领导干部经济责任审计、纠风专项治理、政风行风评议和群众满意度调查，实行了工作日志制度。强化“一岗双责”，严格执纪问责，政府廉政建设得到加强。

　　各位代表，五年的发展成就鼓舞人心，这是市委坚强领导、科学决策的结果，是各级各界同心同德、团结奋斗的结果，凝聚着全市人民的心血和汗水。在此，我代表市人民政府，向全市广大干部群众、各民主党派、工商联、无党派爱国人士，向人民解放军和武警驻泰部队及省以上驻泰单位，向关心支持泰安发展的港澳台同胞、海外侨胞及海内外朋友，表示衷心的感谢！

　　在总结成绩的同时，我们也清醒地看到，经济社会发展中还存在不少困难和问题，政府工作还有许多不足。主要是经济总量偏小，经济下行压力较大；产业结构不合理，部分行业产能过剩，部分企业生产经营困难；发展动力转换较慢，科技创新能力不强，投资拉动效应减缓，内需潜力尚未释放；土地、资源环境、劳动力等要素制约趋紧；扶贫攻坚任务繁重，就业和社会保障压力增大；公共服务体系还不完善，城市管理、社会治理、民生保障等与群众利益密切相关的问题解决还不够好；政府职能转变不到位，作风建设成果不牢固，政府自身建设还有许多工作要做。我们将认真分析原因、找准症结、有的放矢，以更加坚定的信心、更加务实的举措加以解决。

　　“十三五”时期总体要求和目标任务

　　“十三五”时期是全面建成小康社会的决胜阶段。今后五年政府工作总要求是：全面贯彻党的十八大和十八届三中、四中、五中全会精神，以邓小平理论、“三个代表”重要思想、科学发展观为指导，深入贯彻落实习近平总书记系列重要讲话精神，紧紧围绕“五位一体”总体布局和“四个全面”战略布局，牢固树立创新、协调、绿色、开放、共享发展理念，坚持发展第一要务，以提高发展质量和效益为中心，以全面深化改革和实施创新驱动战略为动力，积极适应、把握、引领经济发展新常态，大力实施制造业转型升级、现代服务业繁荣发展、现代农业提质增效、城乡一体协同推进“四轮驱动”战略，加快结构性改革和体制机制创新，扩大对外开放，加强生态建设，保障改善民生，推动经济社会发展实现新跨越，确保与全省同步全面建成小康社会。今后五年的目标任务是：

　　——综合实力显著提升。经济保持中高速增长，在提高发展平衡性、包容性、可持续性的基础上，实现国内生产总值和城乡居民人均收入比2010年翻一番。城乡居民收入增幅超过地区生产总值增幅，农村居民收入增幅超过城镇居民收入增幅。地区生产总值年均增长7.5％以上。

　　——转型发展取得重大进展。创新型城市建设达到更高水平，产业迈向中高端水平，先进制造业加快发展，新产业新业态不断成长，农业现代化水平稳步提高，服务业主导地位初步形成，发展质量效益明显提高。

　　——城乡区域发展更加协调。空间发展格局更加优化，区域互动发展更加融合，城镇服务功能更加完善，主体功能区定位更加明确。泰城中心城市辐射带动能力不断增强，县级城市综合实力和承载力明显提高，新农村建设成效显著，城乡之间、县域之间差距进一步缩小。

　　——文明程度显著提高。中国梦和社会主义核心价值观更加深入人心，公民思想道德素质、科学文化素质、法治素质、健康素质明显提高，文化软实力大幅提升，人民民主更加健全，法治泰安建设全面推进，全社会法治意识不断增强。

　　——人民生活殷实富裕。实现现行标准下贫困人口全部脱贫，就业、教育、文化、体育、社保、医疗、住房等公共服务体系更加健全，基本公共服务均等化水平稳步提高。人民群众生活质量、健康水平、居住环境明显提升，权益得到切实保障，幸福感获得感明显增强。

　　——生态环境优美宜居。污染治理和生态修复实现重大突破，能源资源开发利用效率大幅提高，城乡绿色空间显著增加，林木绿化率稳步提高，生产方式和生活方式绿色、低碳水平不断上升，生态服务功能和生态产品供给能力明显增强。

　　——开放水平全面提升。高水平引进来与大规模走出去实现新突破，利用外资和对外投资、进出口贸易规模效益不断提升，开放型经济发展水平全面提高，经贸合作、文化交流更加密切。

　　——体制机制更加完善。重要领域和关键环节改革取得决定性成果，改革红利加快释放，经济社会发展充满活力。创新社会治理工作取得新进展，社会有序安定和谐。

　　力争到2020年，把泰安建设成具有较强经济综合实力、核心竞争力、文化软实力和生态承载力的经济文化强市、生态宜居城市和国内外著名旅游目的地城市。

　　各位代表，“十三五”规划蓝图已经绘就，我们的发展任务非常艰巨，发展道路仍不平坦，挑战与机遇相互交织。从国际看，既面临着金融危机深层次影响在相当长时间依然存在、不确定因素增多的挑战，也有世界经济在深度调整中曲折复苏、新一轮科技革命和产业变革蓄势待发的机遇。从国内看，既面临着诸多矛盾叠加、风险隐患较多的挑战，也有经济发展方式加快转变、新的增长动能正在孕育、结构性改革积极推进重要战略机遇。从全市看，近年来特别是“十二五”以来，我市历经各类风险挑战，坚持抓项目建设不放松，持续加大转调创力度，发展基础更加牢固、韧劲更加充足。只要我们准确把握重要战略机遇期内涵的深刻变化，牢固树立五大发展理念，加快工作指导转变，永葆豪迈奋进气概，全面建成小康社会目标一定能够如期实现！

　　2016年工作总体安排

　　今年是全面建成小康社会决胜阶段开局之年，是推进结构性改革攻坚之年，也是我市经济转型发展关键之年，做好今年工作意义重大。按照市委总体安排部署，今年主要预期目标是：生产总值增长7.5%—8%；地方一般公共预算收入增长8.5%左右；固定资产投资增长12%左右；社会消费品零售总额增长10.5%左右；进出口实现稳定增长，实际到账外资增长8%左右；城镇居民人均可支配收入增长8%左右，农村居民人均可支配收入增长9%左右；城镇新增就业5万人，城镇登记失业率控制在3.5%以内；人口自然增长率控制在6.9‰；全面完成省政府下达的节能减排降碳等约束性指标。为实现上述目标，我们将紧紧围绕结构性改革，重点抓好以下六个方面工作。

　　（一）更加注重创新驱动发展，下大气力推进结构性改革。坚持“增减”并举，优化存量，提升增量，补齐短板，扩大有效供给，推进产业转型升级、迈向中高端水平。

　　大力推动现代农业提质增效。落实藏粮于地、藏粮于技战略，开展耕地轮作休耕试点，保持粮食种植面积和产量稳定。支持发展优质高效特色农业，抓好国家级、省级“一村一品”示范村镇建设；着力发展优质粮油、有机蔬菜、健康畜禽、生态林果四大主导产业，做大做强水产、桑蚕、茶叶、苗木花卉四大特色产业，推进“泰山四大名药”基地建设和产业化开发。发展精致农业，促进粮经饲统筹、农林牧渔结合、种养加一体、一二三产业融合。积极推广水肥一体化、清洁生产技术，发展生态循环、节能环保、观光休闲型农业。加大优良农作物、林果等新品种开发引进力度。积极培育农民合作社、家庭农场、专业大户等新型经营主体和服务主体，建立利益联结机制，扩大土地托管等服务。发展电子商务与实体流通相结合的物流体系，培育一批特色电商镇、电商村。持续抓好农产品质量安全县创建活动。加大农田水利、农业机械化、农业科技推广、农村饮水安全、重大动物疫病防控、新型职业农民培训等方面投入力度，全面提高农业综合生产能力。严格落实强农惠农富农政策，维护农民合法权益，调动农民生产积极性。

　　加快发展先进制造业。实施工业转型升级发展规划，重点打造输变电装备、汽车及零配件和特色装备三大特色主导产业，做大新材料、电子信息、生物医药和节能环保四大战略性新兴产业，提升纺织、化工和食品三大传统优势产业，着力构建“343”产业体系。坚定不移做强做大骨干企业，努力打造千亿产业（园）和百亿企业。培育壮大中小微企业，引导企业走“专精特新”之路。全面对接“中国制造2025”，大力推进两化融合，积极发展互联网工业。实施工业强基和智能化提升工程，支持企业技术改造和设备更新，促进信息技术向市场、设计、生产等环节渗透，努力建设一批智能工厂和数字矿山，研制一批智能装备和产品，培育一批车间级智能制造企业，开发一批智能制造新业态，建成一批智能制造服务和应用平台。深入实施质量强市和品牌战略，加大驰著名商标和名牌企业培育力度。通过严格环保、能耗、技术等标准，多措并举化解过剩产能。坚持多兼并重组、少破产清算，妥善做好职工安置工作，引导“僵尸企业”平稳退出。努力降低企业制度性交易成本、税费负担、社会保险费、财务成本、物流成本等，为企业转型升级创造条件。切实抓好企业家培训培养，全面提高企业家素质。

　　发展壮大现代服务业。制定实施好18个重点行业和领域转型升级方案，推动生产性服务业向专业化和价值链高端延伸，生活性服务业向精细化和高品质转变，力争年内服务业占生产总值比重提高1.5个百分点左右。完成旅游业发展总体规划编制和泰山总体规划修编，完善市级旅游发展管理体制；加快市旅游经济开发区、徂徕山汶河景区建设，创建徂徕山汶河国家级旅游度假区；发挥泰山龙头作用，丰富提升旅游景区、旅游项目、旅游产品和旅游诸要素内涵和品质，实施旅游满意度提升工程，加快构建国内外著名旅游目的地体系；推进旅游与其它产业深度融合，发展具有泰安特色的文化旅游、乡村旅游、体育旅游、工业旅游、会展旅游；整合各类宣传资源，加大旅游营销和城市形象推广力度。实施批零餐饮转型升级、家政服务品牌培育、电子商务示范带动、社区商业便民利民“四大工程”。加快大宗交易网络平台、第三方网络支付平台建设，培育一批电商示范企业，发展一批电商产业园区。大力发展现代物流业，扶强做大一批物流企业和物流产业园区。健全会展业发展体制机制，积极发展会展经济。支持发展大健康产业，打响“养老泰安·养生泰山”品牌。加大政策扶持力度，推动文化产业转型升级，构建文化产业发展体系。加快发展研发设计、信息服务、动漫创意等现代服务业，培育服务业新业态新模式，不断增加新的消费供给，培植新的消费热点。落实好公积金使用、信贷、税收等优惠政策，积极消化房地产库存。发展住房租赁市场，培育专业化住房租赁企业。

　　着力提升科技创新能力。以更大的力度和气魄推动大众创业、万众创新，释放新需求，创造新供给，推动新技术、新产业、新业态不断涌现。年内高新技术产业产值占规模以上工业总产值比重提高1.5个百分点左右。加快构建具有泰安特色“塔型”科技创新体系，推进以泰山科学院为实体的“泰山创新谷”建设，尤其要加强与驻泰高校的交流合作，促进更多科技成果就地转化。强化企业创新主体地位，组建泰山技术创新与转化联盟，鼓励企业大力开展自主创新和协同创新，支持优势产业和骨干企业承担国家、省重大科技计划。探索运用股权投资、种子基金、担保资金等方式，做强泰山科技创业资金，带动民间资本投向创新型企业。大力发展高新技术产业，继续实施“1351”工程，努力培植一批科技型领军企业，壮大高新技术优势产业集群。积极发展科技服务业，打造高层次研发服务平台。实施“互联网+”计划，支持基于互联网的产业组织、商业模式、供应链等各类创新。实施“人才—基地—项目”一体化工程，支持引进国家“千人计划”专家、泰山学者、高层次创新人才、产业领军人才和高技能人才。

　　（二）更加注重增加有效投资，坚定不移抓好招商引资和项目建设。坚持“扶控”并举，扶持高效高质高端投资，严控产能过剩和“两高一资”项目建设，提高投资有效性和精准度。重点加强关键领域薄弱环节重大工程建设，着力推进青兰高速（泰安段）、104国道改建、济泰东部高速公路、泰安港东平港区和航道建设、公路安全生命防护等工程项目。围绕重点产业、基础设施、社会民生等领域，筛选120个左右市级重点建设项目进行强力推进。抢抓国家、省出台的推动产业转型升级、设立专项引导扶持基金，以及央企省企拆分重组等政策机遇，确定100个前期推进项目，争取更多项目早开工、早建设。围绕“十三五”规划实施、国家重点扶持领域和产业发展方向，策划100个亿元以上重大项目，建立政府投资项目储备库，编制滚动投资计划，形成持续不断、滚动实施的项目储备机制。转变招商理念和招商方式，注重发挥园区、企业主体作用，加大产业招商、定向招商和精准招商力度，重点引进一批引领型、补链型产业项目和科技型、创新型领军企业。

　　（三）更加注重构建发展新体制，毫不动摇推进各重点领域改革。坚持“放管”并举，通过全面深化改革，完善体制机制，为结构性改革增动力、添活力。加快政府职能转变，继续精简和下放行政审批事项，清理规范中介服务，尽量让群众少进一个门、少盖一个章、少跑一趟路。深化国资国企改革，以管资本为主完善国有资产管理体制，对经营性国有资产实行集中统一监管；完善国有企业法人治理结构，有序发展混合所有制经济。深化财税体制改革，完善预决算公开机制和决算审计制度，健全政府预算管理体系；深入推进“营改增”，规范税收优惠政策，健全税收保障机制；科学界定市以下政府事权，调整市县财政分配关系；将政府债务纳入预算管理，从严控制新增债务。积极推行PPP模式，吸引社会资本参与基础设施投资和运营。实施公务用车制度改革。扎实开展“金融创新年”活动，深化政银企合作，扩大社会融资总量；推进200家以上规模企业规范化公司制改制，加快企业挂牌上市步伐；做优做强小额贷款公司、民间资本管理公司和民间借贷登记机构，推开50家以上新型农村合作金融试点；建设泰安金融商务区，设立市级农商行、10亿元以上融资担保公司、30亿元以上金融控股集团、100亿元左右产业基金；优化金融生态环境，建立地方金融信用信息监管平台，防范化解金融风险。深入推进农村集体经济产权制度改革，全面完成农村土地承包经营权确权登记颁证，开展土地承包经营权和农民住房财产权抵押贷款试点。巩固国有林场改革成果，深化集体林权配套改革。继续推进供销合作社综合改革。

　　（四）更加注重开放融合发展，千方百计提高开放型经济发展水平。坚持“进出”并举，深入实施“开放兴市”战略。创新外贸发展模式，优化外贸结构，推动外贸向优质优价、优进优出转变，重点抓好科技兴贸出口创新基地、外贸转型升级示范基地建设和出口农产品质量安全示范市创建，新增一批机电出口基地企业。落实扶持政策，鼓励企业扩大先进技术设备和关键零部件进口。继续开展“双百”工程，扶持一批外贸骨干龙头企业。着力培育一批高水平国际贸易公司和外贸综合服务平台，积极发展跨境电子商务。创新利用外资方式，利用好香港山东周、知名外商泰山行等活动平台，着力引进世界500强和跨国知名企业，引导外资投向我市主导产业、战略性新兴产业、现代服务业等重点领域，鼓励外资企业通过兼并、收购、参股、控股等形式做大做强。支持有条件的企业参与国际竞争与合作，在境外设立加工企业，建立资源开采基地，建设合作园区。坚持对内对外开放并重，对接国家“一带一路”和中韩自贸区，积极融入京津冀协同发展、长江经济带、中原经济区和省“一圈一带”等重大发展战略。推动各类开发区转型创新发展，加快建设泰安高新区海关综合监管区和青岛保税港区（泰安）功能区。

　　（五）更加注重统筹协调发展，坚持不懈推进生态泰安建设。坚持“城乡”并重，推动以人为核心的新型城镇化，让人民群众生活得更方便、更舒心、更美好。

　　强化规划引领，科学谋划城市“成长坐标”，推动城乡建设与产业发展、土地利用、环境保护等“多规”融合。加强对城市空间立体性、平面协调性、风貌整体性、文脉延续性的规划管控，留住城市特有地域环境、文化特色和建筑风格，彰显历史文化名城魅力。进一步提升城市功能，泰城重点抓好高铁新区、中央商务区等城市片区和大型综合体建设，实施万官大街东段、佛光路、桃花源路、北上高大街、发展大街、博阳路南段等城市道路工程，打通东湖路东段、双龙路北段、黄前西干渠路、迎春路南段、学院路北段5条断头路。启动擂鼓石大街、泰山大街、长城路绿化提升和环山路绿道工程。开展地下空间和海绵城市建设试点，加快泰城燃气老旧管网改造和泰安高新区、高铁新区燃气管网建设，完成泰安国电热网与泰城供热管网贯通。完善污水管网系统，抓好冯庄河、唐庄河污水管网建设。编制泰城河道生态水系规划。实施引黄入泰工程。健全城市长效管理机制，巩固提升国家卫生城市迎审成果。

　　加快县域经济发展，支持县市区发挥比较优势，壮大一批特色主导产业，引导产业向园区集聚、人口向城镇集中。健全农村基础设施投入长效机制，推动城市公共服务向农村延伸覆盖。深化户籍制度改革，促进有能力在城镇稳定就业和生活的农业转移人口举家进城落户，与城镇居民享有同等权利和义务。实施居住证制度，努力实现基本公共服务常住人口全覆盖。实施公交优先发展战略，加快公交运力扩容和线路优化，推进城乡公交一体化。深入开展生态文明乡村行动，编制完成农村新型社区和新农村建设规划，加大农村厕所无害化改造和生活污水处理力度，提升城乡环卫一体化水平。积极创建全国文明城市，加强社会主义核心价值观教育和全社会诚信体系建设，开展群众性精神文明创建活动，努力提高公民文化素养和社会文明程度。

　　完善城乡国土空间发展布局，划定全市生态红线，对环境功能区实施最严格保护。推动“工业绿动力”计划，加快发展新能源，抓好燃煤电厂、锅炉超低排放改造。开展大规模国土绿化行动，巩固提升国家森林城市建设水平。积极创建全国水生态文明城市，加快国家湿地公园建设。继续实施“蓝天工程”，确保大气质量持续改善。推动生态示范创建，建成一批省级以上生态村、生态镇、生态县。全面繁荣生态文化，努力形成绿色发展方式和生活方式。

　　（六）更加注重保障改善民生，竭心尽力提高群众幸福指数。坚持“点面”并举，加大民生投入，兜住民生底线，建设和运用好12345民生服务平台，认真解决好群众最关心、最现实的利益问题，让人民群众生活得更有质感、更有尊严、更加安全。

　　实施精准扶贫、精准脱贫“五个一批”工程，加快东平湖移民避险解困工程项目建设，确保完成年度脱贫目标任务。实施更加积极的就业政策，加强就业指导帮扶，促进就业质量提高、总量扩大，尤其突出抓好高校毕业生、复退转业军人、农民工、产能过剩行业失业人员等重点群体就业；鼓励建设创业大学、创业孵化基地、创业园区和创客空间。统筹推进各项社会保险改革，实施社会保险全民参保登记计划，落实城乡居民大病保险制度。提高城乡低保、五保供养、社会救助水平，推动医疗卫生事业与养老服务业融合发展，建成山东泰山社会福利中心和泰安残疾人康复中心。继续推进棚户区和老旧小区改造。

　　坚持教育优先发展，全面推进基础教育综合改革，建设现代学校制度，着力解决城镇中小学“大班额”问题；保障进城务工人员随迁子女平等接受义务教育，做好家庭经济困难学生资助工作；加快建设泰安一中新城校区，确保年内招生；积极发展职业教育、继续教育、特殊教育，鼓励社会力量多种形式兴办教育；大力支持驻泰高校改革发展。全面推进公立医院综合改革，实施分级诊疗制度；落实全面两孩政策，促进人口均衡发展；加强重大疾病综合防治，促进中医药事业发展。健全现代公共文化服务体系，建设数字农家书屋，推动全民阅读；实施泰山文化传承工程和文化遗产保护工程。大力发展体育事业和体育产业。深入开展民族团结进步创建活动，依法管理民族宗教事务。认真抓好国防动员、人民防空、民兵预备役、双拥共建等工作。繁荣发展其他社会事业。

　　扎实开展“七五”普法依法治理工作。深化社区治理创新，加快构建多元共治的现代化社区治理体系。创新社会组织管理，推进政府购买服务。建立现代警务运行机制，加强立体化社会治安防控，抓好矛盾纠纷排查调处。健全应急事件预防预警和应急处置体系，提高应急管理和处置能力。加强食品药品监管，推进“食安泰安”品牌创建。深入开展“企业安全生产责任全面落实年”活动，努力消除各类安全事故隐患。

　　各位代表，功崇惟志，业广惟勤。全面建成小康社会目标任务，对政府自身建设提出了新的更高要求。我们将进一步增强忧患意识、紧迫意识、责任意识，牢牢把握时代脉搏，努力提高驾驭复杂局面和推动发展的能力。一是大力弘扬勇于创新、主动作为的担当精神。引导广大干部适应新常态、学习新知识，加强对五大发展理念、供给侧结构性改革等重大问题、重点任务的研究把握，把上级决策部署与泰安实际紧密结合，创造性地谋划好、落实好发展的“路线图”和“时间表”。始终坚持问题导向，破除思维定势，勇于探索实践，见真着实解决好制约泰安发展的短板和障碍。坚决摒弃小成即满、小富即安，甚至未成也满、未富也安思想，提升发展标杆，勇于赶超先进，争做改革发展的先行者。始终保持奋发有为、积极作为的工作激情，发扬“泰山石敢当”精神，敢担当、愿担当、会担当，争做敢于担责、敢于拼搏、敢于突破的表率。二是大力弘扬真抓实干、狠抓落实的严实精神。按照“三严三实”要求，始终把改革发展当作第一追求、第一责任，沉下心思去谋事，扑下身子去做事，千方百计干成事。尤其是对事关发展大局的重点工作、薄弱环节和具体事项，以钉钉子的精神，咬定目标不放松，锲而不舍抓落实。进一步健全完善目标任务责任制，创新科学发展综合考核工作，加大督查调度力度，坚决纠正不作为、慢作为、乱作为等庸政懒政行为，确保各项工作有部署、有落实，确保各项决策落地生根、取得实效。三是大力弘扬科学决策、尊崇法律的法治精神。牢固树立宪法法律至上意识，深入落实依法治市要求，自觉运用法治思维和法治方式解决问题、化解矛盾、推动工作，切实把政府各项工作纳入法治轨道。大力推进政务公开，健全公众参与、专家论证、风险评估、合法性审查和集体讨论决定的决策机制，严格决策责任追究，推进行政决策科学化、民主化、法治化。自觉接受人大及其常委会监督，定期报告工作。主动加强同人民政协民主协商，大力发展基层协商民主。切实做好人大代表建议和政协提案办理工作。四是大力营造一心为民、风清气正的发展环境。牢固树立群众观念，为民用权，秉公用权，带着感情设身处地考虑群众利益，努力让人民群众过上更好生活。坚持为民务实清廉，驰而不息纠正“四风”。认真执行新修订的中国共产党廉洁自律准则和纪律处分条例，坚决把党的政治纪律和政治规矩挺在前面。全面落实党风廉政建设责任制，深入推进惩治和预防腐败体系建设，完善领导干部经济责任审计制度，教育引导政府工作人员心存敬畏、公私分明，干净用权、清白干事，努力塑造崇廉尚实、干事创业政府良好形象。

　　各位代表，全面建成小康社会的愿景美好而繁重，让我们紧密团结在以习近平同志为总书记的党中央周围，在中共泰安市委的坚强领导下，解放思想，攻坚克难，锐意进取，携手向着第一个百年目标奋勇前进，共创泰安更加美好的明天！
